# A randomized wait-list controlled trial to investigate the role of cognitive mechanisms in parenting interventions on mothers with substance use disorder

**DOI:** 10.1186/s13063-022-06420-8

**Published:** 2022-07-23

**Authors:** Alessio Porreca, Alessandra Simonelli, Pietro De Carli, Lavinia Barone, Bianca Filippi, Paola Rigo, Marinus H. van IJzendoorn, Marian J. Bakermans-Kranenburg

**Affiliations:** 1 Department of Developmental and Social Psychology, University of Padua, Padua, Italy; 2 Department of Psychology, University of Milano-Bicocca, Milan, Italy; 3 Department of Brain and Behavioral Sciences, University of Pavia, Pavia, Italy; 4 Research Department of Clinical, Education and Health Psychology, Faculty of Brain Sciences, UCL, London, UK; 5 Faculty of Behavioural and Movement Sciences, Educational and Family Studies, Vrije Universiteit Amsterdam, Amsterdam, The Netherlands

**Keywords:** Parenting interventions, Substance use disorder, Sensitive parenting, Sensitive discipline, Attentional disengagement, Inhibitory control, Reflective functioning

## Abstract

**Background:**

Maternal substance use disorder (SUD) represents a risk condition for quality of parenting and child development. The current literature highlights the need to identify interventions that effectively enhance the quality of parenting and to better understand which mechanisms are involved in the process of change. The present study protocol describes a randomized wait-list controlled trial that aims to examine (1) the efficacy of the Video-feedback Intervention to promote Positive Parenting and Sensitive Discipline (VIPP-SD) in improving the quality of parenting (i.e., sensitive parenting and sensitive discipline) in mothers with SUD, (2) whether the intervention affects parental cognitive mechanisms (i.e., attentional disengagement to infant negative emotions, inhibitory control confronted with children’s affective expression, and parental reflective functioning), and (3) whether changes in these processes act as mechanisms of change, mediating the effect of the VIPP-SD program on quality of parenting. Moreover, the study aims (4) to explore whether the VIPP-SD has an effect on parenting stress and (5) to compare mothers with SUD to low-risk mothers on the outcome measures.

**Methods:**

The study will involve 40 mothers with SUD and 20 low-risk mothers of children aged between 14 months and 6 years old. Mothers in the SUD group will be randomly divided into two groups, one receiving the intervention (SUD experimental group) and one undergoing treatment as usual (SUD control group). All the mothers will be assessed pre-test and post-test. Quality of parenting will be assessed through observed parenting behaviors, whereas parental cognitive mechanisms will be assessed through neuropsychological tasks and self-report measures.

**Discussion:**

The results of the study will reveal whether an intervention that has been proven effective in other at-risk samples is also effective in improving parenting behaviors in the context of SUD. The results will also provide insight into potential cognitive mechanisms involved in the process of change.

**Trial registration:**

ISRCTN registry ISRCTN63070968. Registered on 25 June 2021. Retrospectively registered

**Supplementary Information:**

The online version contains supplementary material available at 10.1186/s13063-022-06420-8.

## Background

Quality of parenting plays a fundamental role in infant development [1] and is likely to be severely compromised in the context of maternal substance use disorder (SUD [2];), a clinical condition highly associated with dysfunctional parenting practices that in their most severe forms could lead to child maltreatment [3]. Several authors point to the importance of implementing interventions that besides focusing on the condition of drug abuse or dependence per se also target parental functioning, given that recovery from the first is not necessarily associated to improvements in the latter [4–7]. Moreover, a wide array of studies show the relevance of SUD-related cognitive impairments, as for example deficits in attention, memory, decision-making, and problem solving [8–10], to better understand which mechanisms could mediate the effect of interventions [11, 12]. In this regard, much work has been done with respect to adults with SUD (e.g. [13]), but little is known about how such cognitive impairments relate to parenting and parenting interventions in adults with SUD [7].

The present study protocol describes a randomized wait-list controlled trial in which we aim to examine the efficacy of the Video-feedback Intervention to promote Positive Parenting and Sensitive Discipline (VIPP-SD [14];) in improving the quality of parenting in mothers with SUD. Secondly, the study aims to investigate whether the intervention affects parental cognitive mechanisms, improving attentional disengagement to infant negative emotions, inhibitory control in front of children’s affective expression, and maternal reflective functioning. Finally, the study aims to detect whether changes in parental cognitive mechanisms play a role in mediating the effect of the VIPP-SD program on the quality of parenting measured through observed parenting behaviors. To accomplish this, we will compare mothers with SUD receiving the intervention (SUD experimental group) to mothers with SUD undergoing treatment as usual (SUD control group). Secondly, we will compare mothers with SUD to low-risk mothers recruited from the general population, to see whether the intervention reduces expected differences between mothers with SUD and low-risk mothers. In the following paragraphs, rationale, objectives, hypotheses, and methods of the study are presented, followed by a discussion of the possible implications.

### Quality of parenting in mothers with SUD

#### Observed parenting behaviors

Observed parenting behaviors constitute a key access to the quality of parenting in infancy and childhood, providing a measure of the parent’s ability to take care of the child and of the child’s actual experience of care [15–19]. Maternal SUD jeopardizes the quality of parenting behaviors, affecting in multiple ways sensitive parenting [15, 17, 20] and parental sensitive discipline [21, 22], two facets of positive parenting associated to favorable developmental outcomes in children [23, 24]. Compared to low-risk populations, mothers with SUD show less optimal sensitivity and responsiveness to children’s emotional signals [25–29] and are more inclined to be hostile, directive, and interfering with their activities [30–34]. These negative parenting behaviors have been linked to unfavorable outcomes in offspring, as insecure and disorganized attachments [35], and a higher risk to be involved with child protective services [36]. Moreover, mothers with SUD are more inclined to adopt negative disciplinary strategies [37], ranging from the use of harsh discipline [38–40] to the adoption of laissez-faire, characterized by withdrawal and lack of limit-setting, and role reversal [41, 42]. These practices tend to be ineffective and are related to several undesired developmental outcomes in children, including internalizing and externalizing problems [43, 44]. Therefore, parenting behaviors constitute one of the main targets of parenting interventions in the condition of SUD [45, 46]. In the present study, we aim to test the efficacy of the VIPP-SD, a short-term evidence-based parenting intervention based on attachment theory [17, 47] and social learning theory [48, 49], in improving positive parenting strategies in a clinical population of SUD mothers with young children. We will measure sensitive parenting and sensitive discipline using observational scales (the Emotional Availability Scales [50] and a scale for harsh discipline [51]) under various structured conditions (free-play and two compliance tasks), comparing mothers with SUD receiving the intervention to mothers with SUD undergoing treatment as usual (TAU) and to low-risk mothers. According to previous studies, we expect that (1) the quality of observed parenting behaviors in SUD mothers is higher after the intervention compared to the randomized control group receiving TAU and (2) mothers with SUD will, at pretest, show poorer parenting behaviors compared to the low-risk group.

### Cognitive mechanisms involved in parenting

#### Attentional mechanisms: attentional bias to child negative emotions

Attentional processing of child stimuli provides one of the basic cognitive mechanisms for sensitive parenting. Human adults present a selective bias in the processing of infant faces, which is of potential evolutionary value [52, 53] since it facilitates a detailed screening of facial mimicry and increases the likelihood of appropriate responses [54]. It has been shown that mothers present a preferential attentional bias for child negative emotions, finding it more difficult to disengage their attention from visual stimuli displaying sadness or distress [55, 56]. This process is attenuated in the presence of psychopathology and high parenting stress [53, 57] and has proved to be sensitive to treatment [54]. We hypothesize this same attentional mechanism to be disrupted in mothers with SUD, given the high incidence of parenting stress [58], psychopathology [59, 60], and the overlap between brain reward regions associated to substances and to infant-related stimuli [61, 62]. This hypothesis is further supported by studies that provide evidence for disruptions in normative attentional processes in the context of substance dependence [63, 64].

In the current study, we aim to test whether attentional bias to child face in mothers with SUD, measured with a computerized neuropsychological task, is enhanced through the application of the VIPP-SD. According to previous studies, we expect that (1) attentional bias to child negative emotions in mothers with SUD increases after the intervention compared to the randomized control group receiving TAU and (2) at pre-test, mothers with SUD will show lower attentional bias to child negative emotions compared to the low-risk group.

#### Inhibitory mechanisms: inhibitory control when exposed to child emotions

Inhibition of parenting negative strategies represents another basic cognitive mechanism of sensitive parenting [65]. At the cognitive level, the inhibition of non-optimal responses relies on inhibitory control, a “lower order” component of executive functions responsible for the regulation of attention, thoughts, or behaviors, according to internal and external stimuli [66–69]. Chronic drug use is associated with severe frontal and prefrontal cognitive dysfunctions which result in the inability to inhibit dominant behavioral responses activated by craving, which lead to the search and assumption of drugs [70–72]. Impairments in prefrontal activity are responsible for the onset and maintenance of substance dependence [70–75] and have been found in mothers with SUD also when observing infants displaying different emotional expressions [62, 76].

Traditionally, inhibitory control has been investigated through go/no-go paradigms, which involve the presentation of stimuli, alternating go conditions, where the individual has to respond to the displayed cue, and no-go conditions, where the individual has to inhibit their response [66, 77]. In the current study, we aim to test whether inhibitory control when exposed to child emotions in mothers with SUD, measured through a computerized emotional go/no-go task, improves after the administration of the VIPP-SD. We expect that (1) inhibitory control when exposed to child emotions in mothers with SUD will increase after the administration of the intervention compared to the randomized control group receiving TAU and (2) compared to the low-risk group, mothers with SUD at pre-test will show lower inhibitory control when exposed to child emotions.

#### Maternal reflective functioning

Reflective functioning describes the parents’ ability to reflect upon their own and their children’s experience and behaviors in terms of mental states [78–80]. This mechanism is associated with positive and negative parenting strategies [79, 81, 82] and with children’s use of their mothers as secure base [83]. Mothers with SUD present difficulties in emotion regulation [84] as well as poor reflective functioning abilities, with the risk to develop negative, idealized, or fragile representations of their children and their parental role [46, 85, 86]. Postnatal levels of mentalizing abilities have been identified as predictors of clinical prognosis in the context of substance abuse [87], and reflective functioning represents an important target of parenting interventions within this clinical population [46, 87]. Improvements in reflective functioning in response to parenting interventions have been associated with increases in quality of observed parenting behaviors and with improvements in children’s regulation [7, 85]. For the purposes of the present study, we will examine self-reported maternal reflective functioning pre- and post-intervention. We expect that (1) reflective functioning in mothers with SUD improves after the intervention compared to the randomized control group receiving TAU and (2) mothers with SUD at pre-test present lower reflective functioning abilities with respect to the low-risk group.

#### Other variables relevant in the context of maternal SUD

Given the complexity of the SUD condition, especially in mothers, various domains of adult functioning will be included as control variables in the present study. A brief description and rationale for each one is reported below.

##### Parenting stress

Mothers with SUD are more likely to experience high levels of stress in their caregiving role, much of which is dependent on the condition of substance addiction and related risk factors, such as health conditions and psychosocial difficulties [58, 62, 88, 89]. The reiteration of substance use in time decreases the salience of infant-related stimuli that become less rewarding for mothers and risk to be perceived as a source of additional stress rather than part of a mutually fulfilling system [62, 90]. High levels of parenting stress have been often linked with difficulties in providing high-quality parenting and are associated with hurdles in mother-child interactions, lack of parental warmth, and increases in harsh parenting [91]. For the purpose of this study, we will measure parental stress using the short form of the Parenting Stress Index [92] before and after the intervention, expecting a decrease in levels of stress after treatment.

##### Maternal psychopathology

Individuals with SUD present an increased risk for psychopathology and psychological maladjustment [93, 94], which has been linked to poor executive functioning [95] and to several adverse treatment outcomes, such as increased severity in individual maladjustment and early relapse to substance use [96]. The presence of psychopathological symptoms in mothers with SUD represents an additional risk factor for caregiving practices, exacerbating difficulties experienced during mother-child interactions and increasing the risk to adopt negative parenting strategies [97–99]. In the present study, we will control for the potential confounding role of maternal psychopathology when examining the efficacy of the VIPP-SD. Maternal psychopathological symptoms and psychological distress will be measured pre- and post-treatment and considered as a confounder.

##### General executive functioning

Inhibitory control represents a “lower order” component of executive functions and, together with working memory and cognitive flexibility [66, 100, 101], allows for the activity of “higher order” executive functions, such as reasoning, problem solving, and planning [66, 102, 103]. A wide array of research has highlighted that individuals with chronic and heavy substance abuse present with damages to executive functioning, which could act as predisposing, retention, and relapse factors for substance assumption [11–13, 104–106]. Moreover, research on parenting found some associations between executive functions and parental practices [68, 69]. In the present study, we will control for the potential confounding role of general executive functioning to investigate the specific impact of VIPP-SD on parental inhibitory control when exposed to child emotions and to ascertain its role as a mechanism of change in parenting behaviors. Mothers’ general executive functioning will be measured pre- and post-treatment and its residual score after taking into account its overlap with inhibitory control will be used as a potential confounder in statistical analyses.

### Intervention

For the purpose of the current study, we selected as eligible treatment the VIPP-SD [14, 107], an evidence-based intervention aimed at enhancing parental sensitivity and sensitive discipline in parents of toddlers and preschool children. The protocol has proven to be effective in different randomized controlled trials in various populations [108–110], with a recent meta-analytic study reporting a combined effect size of *d* = 0.47 [21], and its characteristics appear particularly suitable for mothers with SUD.

Specifically, the limited number of sessions, the focus on interactive behaviors, and the home-based nature of the protocol are likely to help maintain the mothers’ engagement, limiting the risk of dropouts before the end of treatment [111–113]. The ease of understanding of the contents supports comprehension of the intervention themes, preventing dropout risks linked to deficits in attention and cognitive functioning [11, 13]. The use of the video-feedback technique, the focus on child signals [45], and the provision of information on child development could help mothers to adapt their interaction to the child’s age-appropriate level [114, 115]. These features support the feasibility of applying the intervention to this specific clinical population, offering the possibility to administer it both in the context of outpatient and inpatient conditions.

### Aims and hypotheses

**Fig. 1:**
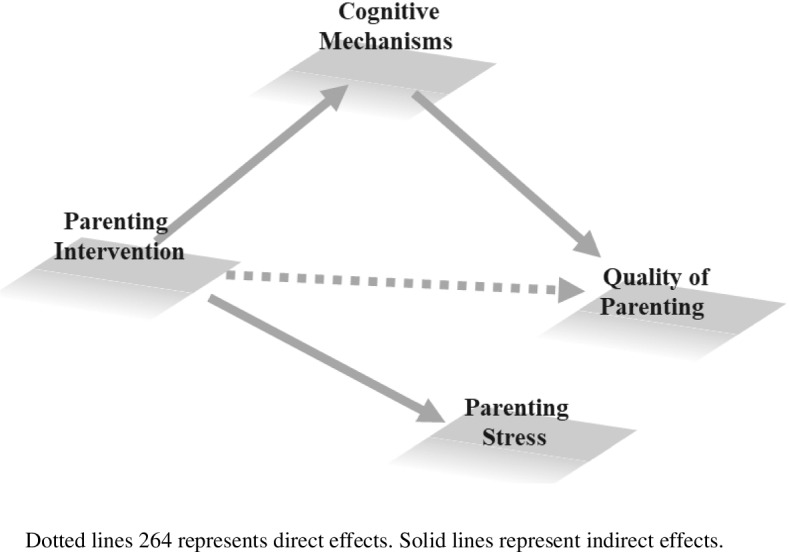
Aims of the study and hypothesized mechanisms of change

1. Our primary aim is to investigate intervention effects on the quality of parenting measured through observed parenting behaviors, in mothers with SUD. We will investigate whether the intervention affects sensitive parenting and gentle but consistent discipline. We expect that, from pre-test to post-test, the quality of observed parenting in the SUD experimental group increases more or decreases less than in the SUD control group.
2. Our secondary aim is to investigate intervention effects on parental cognitive mechanisms. We will investigate whether the intervention affects the mothers’ performances on (2a) an attentional bias reaction time paradigm aimed at measuring attentional bias to negative emotions and on (2b) a go/no-go task involving child faces displaying different emotions aimed at measuring inhibitory control in response to child emotions, respectively. Moreover, we will investigate whether the intervention affects (2c) self-perceived reflective functioning. We expect that the intervention modifies the mothers’ performances on the two tasks and self-reported reflective functioning in the SUD experimental group with respect to the SUD control group.
3. Our tertiary aim is to investigate whether changes in parental cognitive mechanisms affect intervention effect on parenting. Specifically, we will investigate whether changes in the mothers’ (3a) attentional bias, (3b) inhibitory control, and (3c) reflective functioning account for changes observed in parenting. Specifically, we expect that improvements in the mothers’ performances on the cognitive measures will be associated to improvements in the quality of observed parenting in the SUD experimental group (Fig. 1).
4. Our fourth objective is to explore whether the administration of the intervention has an effect on parenting stress. Specifically, we expect that, after the administration of the VIPP-SD, perceived parenting stress in the SUD experimental group decreases compared to the SUD control group.
5. Our fifth objective is to compare the SUD experimental group and the SUD control group to the low-risk group with respect to post-test measures.

## Methods/design

### Study design

The study is a randomized wait-list controlled trial aimed at investigating the role of parental inhibitory control, attentional disengagement, and reflective functioning in the efficacy of the VIPP-SD, an evidence-based parenting intervention, in mothers with SUD. The protocol was developed in line with the SPIRIT guidelines ([116], see Additional files 1 and 2). The project will involve a group of mothers with SUD and a group of low-risk mothers. The intervention will be randomly delivered to half of the mothers in the SUD group, through a wait-list approach. Specifically, mothers from the SUD group will be randomly assigned to one of two conditions: (1) an experimental condition (SUD experimental group), treated with the VIPP-SD, and (2) a wait-list condition (SUD control group), with TAU. Participants in both groups will be assessed pre- and post-treatment/wait-list for primary and secondary outcome measures. Participants in the SUD experimental group will be reassessed at a 2-month follow-up, to see whether outcomes measured during the post-test phase remain stable, whereas at the end of the post-test phase participants in the SUD control group will be administered the intervention.

**Fig. 2:**
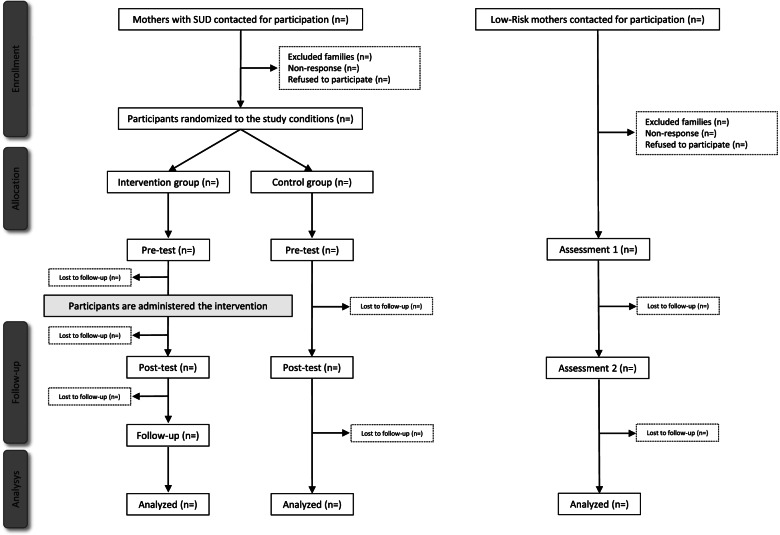
Diagram of participants’ flow through the trial

Mothers in the low-risk group will undergo two measurements (respectively assessment 1 and assessment 2) where we will collect the same measures collected in the SUD group (quality of parenting behaviors, attentional disengagement, inhibitory control, reflective functioning, parenting stress, maternal general executive functioning, and psychopathology). The two measurements in the low-risk group are scheduled at a 3-month distance, a time frame that equals the length of the VIPP-SD, and will serve as a comparison for the pre-test and the post-test phase of the SUD experimental group. Figure 2 presents the diagram of participants’ flow through the trial.

### Participants

#### Recruitment

The project will involve 60 mothers with toddlers and preschool children, aged between 12 months and 6 years old. The recruitment will be carried out in northern Italy. The SUD group (*n* = 40) will be composed of women with a history of SUD diagnosis recruited through residential and outpatient facilities that treat SUD and other psychiatric disturbances. History of SUD diagnosis is defined as severe substance use, abuse, or dependence, within 2 years preceding enrollment, with respect to one or more substances of interest (e.g., alcohol; caffeine; cannabis; hallucinogens; inhalants; opioids; sedatives, hypnotics, or anxiolytics; stimulants; tobacco; and other or unknown substances) according to criteria outlined by principal diagnostic manuals [2]. Studies on SUD identified a rate of dropout ranging from 23 to 50% in outpatient treatment [117, 118] and from 17 to 57% in inpatient treatment [119, 120]. In order to prevent attrition bias, and to reach the expected number of participants completing the trial, we will oversample participants in the SUD group by 20%. The low-risk group (*n*=20) includes mothers recruited from the general population. Mothers in the low-risk group should present the absence of history of SUD diagnosis and absence of history of treatment in residential and outpatient facilities that treat SUD and other psychiatric disturbances. We will screen them using a specific checklist administered at their enrollment. Inclusion criteria for each group foresee that mother and child live together or in close contact (at least 4 times per week) at the time of recruitment and during the various stages of the study. Exclusion criteria concern the presence of diagnosed psychotic disorders in an active phase, organic brain disorders that prevent the execution of the tasks, and child developmental pervasive disorders. Mothers in the SUD group will be contacted through the help of healthcare providers, whereas mothers in the low-risk group will be contacted through family centers, nursery schools, and academic database sources. Potential candidates will be invited to take part in the study and will receive more information and asked informed consent when they agree to participate. Participation in the study is free.

#### Randomization

Randomization of the SUD group to the SUD experimental and SUD control group will be carried out a priori and each participant will be assigned to a predetermined condition once enrolled in the study. We will adopt a blocked randomization with randomly selected block sizes, stratified with respect to child’s gender and age. Blocked randomization enhances the chance that treatment groups are equal in size and uniformly distributed according to key outcome-related characteristics [121]. Randomization will be implemented charging three distinct figures of sequence generation, participant enrollment, and participant allocation [122]. Sequence generation will be carried out by an independent researcher through R [123], with a 1:1 allocation using random block sizes of 2 and 4, stratified by gender and child age (younger vs older than 30 months). The researcher performing sequence generation will not be involved in recruitment, intervention administration, data collection, or data coding. To guarantee allocation concealment and to avoid selection bias, an independent researcher not involved in the trial will be charged to the custody of the sequences generated and to the assignment of the participants to each condition. Once the participants have been recruited and assessed for the pre-test phase, the researcher will be contacted by phone and hear the allocation of each individual.

#### Sample size and power

The sample size for the study was calculated based on power, level of significance, and size of the treatment effect expected [124], referring to previous study reporting on RCTs addressed to mothers with SUD [86]. Power was set at the conventional level of 80%. Regarding the size of the expected treatment, recent meta-analytic work testing the effectiveness of VIPP-SD on sensitive parenting yielded a combined effect size of *d* = 0.47, indicating that the intervention significantly increases sensitive caregiving [21, 125]. For the purposes of the present study, a statistical power analysis was performed for sample size estimation (repeated measures ANOVA within-between interaction, G*Power 3.1.9.2). For our primary, secondary, and fourth aims, testing the effect of the intervention on parenting and on parental cognitive mechanisms through repeated measures analyses with *α* = .05, a statistical power (1 − *β*) = 0.80, and a medium-sized effect of *d* = 0.47, a minimum sample size of *n* = 34 is required. For our third and fifth aims, testing mediating mechanisms, the proposed sample size ensures a power >80% since the power to detect mediating effects is generally larger than it is for main effects [126]. Considering the high risk of attrition in the SUD group, we will adopt oversampling strategies when managing missing data [127] and intention-to-treat analyses [128], in order to preserve statistical power.

### Intervention

#### Intervention group

Participants in the SUD experimental group will be administered the VIPP-SD, an evidence-based intervention that adopts the technique of video-feedback for enhancing (1) sensitive parenting and positive parent-child relationships and (2) sensitive discipline, reducing children’s emotional and behavioral problems [21]. The protocol is based on attachment theory [17, 47] and on social learning theory [48, 49]. The intervention is manualized and delivered in 6 sessions, each one affording themes relevant for sensitive parenting and sensitive discipline. The manual describes the structure, the themes, and the exercises suggested to parents during the different sessions [21]. Each home visiting begins with the videotaping of structured parent-child interactions, which are then reviewed together with the intervener during the following sessions. Between sessions, the intervener prepares comments for each video fragment to-be-seen according to the themes that in each meeting are salient for sensitive parenting and sensitive discipline (see Table 1). Themes for sensitive parenting concern the difference between exploration versus attachment behavior, the technique of speaking for the child, and the importance of sensitivity chains and of sharing emotions during parent-child interaction. Themes inherent sensitive discipline concern inductive discipline and distraction, positive reinforcement, sensitive time-outs, and empathy for the child [14, 21]. The first four sessions introduce these relevant topics whereas the two last sessions serve as booster sessions to revise and integrate the themes previously afforded and to reinforce new acquisitions in the parent [21]. The VIPP-SD has been applied to different settings [129–131], including residential facilities [132], and has been successfully adopted with different clinical and at-risk groups [133–135]. Previous meta-analytic work provided evidence for a substantial effect of VIPP-SD in enhancing positive parenting [21]. Treatment will be administered by a group of interveners officially trained and showing fidelity of treatment. Treatment fidelity will be guaranteed through continuous supervisions of the interveners with the fourth author, an official Italian trainer on the method.

**Table 1:** Themes relevant for the VIPP-SD

Session	Sensitive parenting	Sensitive discipline
1.	Exploration vs. attachment behavior	Inductive discipline and distraction
2.	Speaking for the child	Positive reinforcement
3.	Sensitivity chain	Sensitive pause
4.	Sharing emotions	Empathy for the child
5.	Booster session	Booster session
6.	Booster session	Booster session

Retrieved from Juffer et al. [21]

#### Wait-list group

The experimental SUD group will be administered the VIPP-SD immediately after the pre-test phase whereas, during this period, participants in the SUD control group will undergo TAU which involves individual and group psychotherapy, psychopharmacological treatment, and educational intervention. After the post-test phase, the intervention will be delivered also to the wait-list group.

### Measures

#### Primary aim

Our primary aim is to investigate whether the intervention is effective in improving the quality of parenting, measured through observed parenting behaviors operationalized as parental sensitivity and sensitive discipline. According to previous work [51], parental sensitivity will be assessed during free play, whereas sensitive discipline will be assessed during two compliance tasks, a do not touch situation and a clean-up situation. Each procedure will be video-recorded and coded by raters blinded to the aims of the study and to the participants’ condition. The observational procedures will be assessed with the Emotional Availability Scales [50], 6 scales that evaluate the parent’s and the child’s contribution to the interaction. Moreover, sensitive discipline will also be assessed through a scale aimed at assessing the presence of verbal or physical harsh discipline [51]. Multiple trained raters, blinded to the study objectives and to the participants’ condition, will code videotapes. Regular meetings with the developers of the coding systems will be scheduled, to guarantee reaching of sufficient reliability with the coding system and avoiding rater drift. Moreover, inter-rater reliability will be calculated on a subset of the videos.

#### Secondary aim

Our secondary aim is to investigate intervention effects on the mothers’ attentional disengagement to infant negative emotions, inhibitory control in front of children’s emotions, and on reflective functioning. A description of the measures adopted for each mechanism is provided in the following sections.

##### Measurement of attentional disengagement 

Maternal disengagement to infant negative emotions will be measured through an attentional bias reaction time paradigm [57]. During this attentional task, subjects are required to focus on a central go/no-go signal on the computer screen (a green or a red cross). A horizontal and a vertical line are presented as peripheral stimuli at the two extremities of the screen. The red central cross represents no-go trials, where participants are required to press the space bar. The green cross indicates go trials, where the individual is required to localize the position of the horizontal bar and press the appropriate keyboard response (A=left, L=right). During the task, distressed or non-distressed infant faces will appear behind the cross, as background images, slowing down the disengagement of attention. Each trial will begin with a fixation cross at the center of the screen (750 ms), followed by the stimulus display (240 ms, including the go/no-go signal, the face stimuli, and the two peripheral lines), and finally a blank screen until a response is registered. Participants are instructed to ignore pictures appearing in the task. An index of attentional bias towards distressed infant faces will be calculated, computing the difference between mean reaction times (ms) on distressed and non-distressed infant trials for each individual [55, 57].

##### Measurement of inhibitory control 

The mothers’ inhibitory control in front of children’s emotions will be assessed on a computerized go/no-go task involving child faces displaying different emotions (positive vs negative). Visual stimuli are selected from the Child Affective Facial Expression set [136], a validated set of 2- to 8-year-old children’s faces. During the emotional go/no-go task, the participants will be randomly presented with a child showing a positive or a negative emotion. Positive emotions represent go trials, where participants are required to press the space bar. Negative emotions indicate no-go trials, where individuals are required to inhibit their behavior, doing nothing. Each trial will begin with a fixation cross at the center of the screen (2000 ms), followed by the stimulus presentation (500 ms), and a blank screen with a fixation point (1500 ms) for response registration. Each of the 60 faces will be shown once, for a total of 60 trials. Reaction times and accuracy of the performance will be recorded and yield a measure of the mothers’ inhibitory control when exposed to children’s affective expressions.

Given previous studies reporting correlations between selective attention and inhibition of action in normative [137] and addicted individuals [63, 138], a preliminary correlational analysis will be carried out on performance on the two cognitive tasks. When the two measures are correlated, a composite score will be calculated [139, 140]. When not, performances at the two tasks will be considered as separate variables in statistical analyses.

##### Measurement of maternal reflective functioning

Maternal reflective functioning will be assessed using the Parental Reflective Functioning Questionnaire [141], an 18-item self-report measure aimed at investing perceived reflective functioning in parents, intended as curiosity about the child’s mental states, effort/refusal to understand mental states, and how they relate to behavior.

#### Tertiary aim

Our tertiary aim is to investigate whether changes in the mothers’ emotional modulation of inhibitory control, attentional disengagement, and reflective functioning account for changes in the quality of parenting. To do this, we will examine whether changes in the mothers’ performances on the attentional bias reaction time paradigm and on the go/no-go task involving child faces displaying different emotions, as well as maternal self-reported reflective functioning, account for changes in observed parenting behaviors.

#### Fourth aim

Our fourth objective is to explore whether the administration of the intervention has an effect on parenting stress, which will be measured through the Parenting Stress Index – Short Form [92], a 36-item self-report measure aimed at investigating the stress experienced by parents during parental practices.

#### Fifth aim

Our fifth objective is to compare the SUD experimental group and the SUD control group to the low-risk group. To do this, we will compare the post-test measurements of the experimental and control SUD groups with the two assessments in the low-risk comparison group.

### Control variables

When testing changes in the mothers’ attentional disengagement, inhibitory control, and reflective functioning, we will control for the potential confounding roles of general maternal executive functions, and maternal psychopathology. As far as it concerns general executive functions, we will measure inhibitory control, working memory, cognitive flexibility, and planning through standardized neuropsychological tasks involving neutral stimuli. Specifically, we will use a go/no-go task [142], the Corsi Block-Tapping Task [143, 144], a short-form of the Berg Card Sorting Test [145–148], and the Tower of London Test [149–151]. All the tasks are computerized and retrieved from the open source software system PEBL - Psychology Experiment Building Language [152].

Concerning maternal psychopathology, we will administer the Symptom Checklist-90 Revised [153], a 90-item self-report questionnaire designed to evaluate the presence of psychopathology along different symptom dimensions and global distress indexes.

### Statistical analyses

Statistical analyses will be carried out according to intention-to-treat principles [154]. Data distributions will be inspected to check for normality and data transformation will be applied when normality assumptions are violated [155]. Missing data will be inspected to check whether they are missing completely at random, at random, or not at random, and multiple imputation procedures will be applied to manage them [156]. For the primary, the secondary, and the fourth aims, we will first adopt linear mixed models for intent-to-treat analyses and subsequently apply repeated measures models on complete cases. To estimate the intervention effect on parenting and on parental cognitive mechanisms, we will define experimental condition as between-subjects factor and time-point measurements as within-subjects factor. For our tertiary and fifth aims, we will use the Montoya and Hayes approach [157] in a repeated measures design to test whether changes in cognitive mechanisms mediate the intervention effects on parenting.

### Data management and ethics

The study will be carried out in line with national and international standards of good clinical practice. All the participants will be asked written and verbal informed consent and during the entire unfolding of the project participants will be reminded that participation to the study is voluntary and that they have the possibility to withdraw from the study at any time, without consequences. The research protocol received ethical approval from the Ethical Committee of the University of Padua (Protocol: 3475). All data will be managed confidentially and stored on secure drives of the University of Padua. Part of the data could be temporarily stored on drives of the Universities of Pavia, Erasmus University Rotterdam, Vrije Universiteit Amsterdam due to coding, supervision, and statistical analyses. The VIPP-SD has been previously used in a number of studies, including clinical populations, and did not present risks associated with the intervention. No criteria for interrupting the administration of the intervention have been highlighted, except that of the participants’ choice. Authorships for journal articles will be defined according to APA or ICMJE guidelines.

### Ancillary and post-trial care

Given the absence of anticipated harm due to study participation, no specific provision for post-trial or ancillary care is foreseen by the study protocol. Most of the participants in the SUD groups are expected to remain within their TAU after the project. Whenever clinical conditions that require specific attention emerge during study participation, the participants will be instructed to contact their referring clinicians in order to obtain appropriate treatment.

### Protocol amendments

Currently, the study is being implemented in accordance with the procedures described in the present study protocol. Amendments are being submitted for any change in the existing protocol that significantly affects the scopes of the investigation or the scientific quality of the study. The amendment will contain a brief description of the changes and reference to the previous submission (date and number). Approval for any substantial change to the original protocol will be requested by the Institutional Review Board before amendment submission.

### Public and patient involvement

Although not measured systematically, patient and public involvement had a fundamental impact on the initial stages of the development of the study design. Part of the clinical and research expertise in the context of parental SUD has been developed through a long-time partnership with Casa Aurora, Comunità di Venezia scs, a residential facility located in northern Italy that provides treatment to mothers with SUD and other psychiatric disorders. The continuous communication with the staff of the facility and with the patients in treatment guided the setting of the research objectives and the intervention goals, so that they would be relevant for both users and health services. Specifically, in line with professionals’ suggestions, we attempted to design a protocol that could be easy to understand for professionals with different backgrounds (e.g., psychiatrists, psychologists, nurses, and professional educators) and flexible enough to be introduced both in residential facilities and outpatient services, considering variation in organizational dynamics. Furthermore, in response to several mothers’ complaints about difficulties in playing and engaging with their children, or in setting age-appropriate limits, we made the choice for the VIPP-SD program (whose goals are in line with the mothers’ requests). In this sense, during the development of the protocol, we attempted to integrate scientific requests with services and users’ perspectives, to ensure that the research would be appropriate for patients’ and facilities’ needs while still being valid and robust from a scientific point of view [158]. Moreover, public and patient involvement is being achieved during the implementation of the study, collecting professionals’ and patients’ feedback and considerations about various stages of the study.

## Discussion

The present study protocol describes a randomized wait-list controlled trial in which we aim to test the effect of the VIPP-SD in improving quality of parenting and in changing parents’ cognitive mechanisms in the context of maternal SUD. Moreover, the current study aims to test whether changes in cognitive mechanisms account for changes in observed quality of parenting. Testing these hypotheses has a significant impact both from a clinical and an empirical point of view.

Clinically, testing an intervention which is brief, standardized, and effective in improving the quality of parenting and of parent-child relationships could provide an important addition to programs that target parents with SUD. This intervention could be adopted in facilities and by healthcare providers parallel to interventions aimed at reducing substance abuse and parental psychopathology. The specific features of the VIPP-SD (e.g., the focus on positive aspects and on the strengths of parent-child relationships) are expected to support the development of early working alliance with health facility and professionals, which has been pointed out as a consistent predictor of engagement and retention in drug treatment [159]. Moreover, the adoption of such an intervention could sensitize healthcare providers and social workers to the importance of promoting sensitive parenting behavior, which is not only focused on instrumental care and satisfaction of basic needs, but also emotionally attuned to the child’s communications.

From an empirical perspective, the present study could provide further knowledge of the mechanisms underlying observable parenting and important insights into the cognitive mechanisms that could mediate the behavioral effect of interventions.

Strengths of the study are represented by the adoption of a randomized controlled trial design, the collection of behavioral measures (observed parenting behaviors and cognitive tasks), and the use of an evidence-based intervention that has proven effective in previous RCTs in various at-risk samples. Novelty of the study is also the focus on a specific clinical sample, for which the intervention has not been tested yet.

Limitations of the protocol are linked to the use of self-report measures for parenting stress, reflective functioning, and psychopathology and to the heterogeneity of the sample (due to the condition of poli-abuse of substances).

## Trial status

Recruitment began in March 2020 and will end in October 2023.

## Supplementary Information

**Additional file 1.** SPIRIT checklist.

**Additional file 2.** SPIRIT figure.

## Data Availability

Data sharing is not applicable to this article as no datasets were generated or analyzed during the current study.

## Abbreviations

SUD: Substance use disorder
VIPP-SD: Video-feedback Intervention to promote Positive Parenting and Sensitive Discipline
TAU: Treatment as usual
