# Supplementary material for: A randomized wait-list controlled trial to investigate the role of cognitive mechanisms in parenting interventions on mothers with substance use disorder
Source: Trials. 2022 Jul 23;23:588. doi: 10.1186/s13063-022-06420-8 (PMC9308363; doi:10.1186/s13063-022-06420-8)
Supplement: Supplementary file 2 — Additional file 2. SPIRIT figure. [file 13063_2022_6420_MOESM2_ESM.pdf]

	STUDY PERIOD							
	**Enrolment**	**Allocation**	**Post-allocation***					**Close-out**
**TIMEPOINT**	***-t*₁**	**0**	***t*₁**	***t*₂**	***t*₃**	***t*₄**		***t*ₓ**
**ENROLMENT:**								
**Eligibility screen**	X							
**Informed consent**	X							
[List other procedures]	X							
**Allocation**		X						
**INTERVENTIONS****								
[Intervention A]				X				
[Intervention B]				X				
[Intervention C]						X		
**ASSESSMENTS:**								
[Baseline variables]	X	X	X					
[Outcome variables]					X	X	.	X
[Other data variables]			X		X			X

* t_1= pre-test phase, t_2=intervention phase, t_3=post-test phase, t_4=follow-up phase
Intervention A= VIPP-SD to the SUD Experimental Group, **Intervention B**= TAU to the SUD Control Group, **Intervention C**= VIPP-SD to the SUD Control Group
